# Why are RNA virus mutation rates so damn high?

**DOI:** 10.1371/journal.pbio.3000003

**Published:** 2018-08-13

**Authors:** Siobain Duffy

**Affiliations:** School of Environmental and Biological Sciences, Rutgers, the State University of New Jersey, New Brunswick, New Jersey, United States of America

## Abstract

The high mutation rate of RNA viruses is credited with their evolvability and virulence. This Primer, however, discusses recent evidence that this is, in part, a byproduct of selection for faster genomic replication.

RNA viruses have high mutation rates—up to a million times higher than their hosts—and these high rates are correlated with enhanced virulence and evolvability, traits considered beneficial for viruses. However, their mutation rates are almost disastrously high, and a small increase in mutation rate can cause RNA viruses to go locally extinct. Researchers often assume that natural selection has optimized the mutation rate of RNA viruses, but new data shows that, in poliovirus, selection for faster replication is stronger and faster polymerases make more mistakes. The fabled mutation rates of RNA viruses appear to be partially a consequence of selection on another trait, not because such a high mutation rate is optimal in and of itself.

Mutations are the building blocks of most of evolution—they are the variation upon which natural selection can act, and they are the cause of much of the novelty we see occur in evolution [1]. However, most mutations are not beneficial for the organisms with them. Many mutations cause organisms to leave fewer descendants over time, so the action of natural selection on these mutations is to purge them from the population. While a small percentage of mutations are helpful and some are inconsequential (neutral or nearly neutral in effect), a large portion of mutations are harmful [2]. While the fraction of mutations that are harmful versus beneficial may change in different organisms, in different environments, and over time, deleterious mutations are thought to always outnumber beneficial mutations [2]. That remains true whether an organism has a low mutation rate or a high mutation rate, and biological entities differ dramatically in their per-nucleotide mutation rate (over eight orders of magnitude, Fig 1).

**Fig 1:**
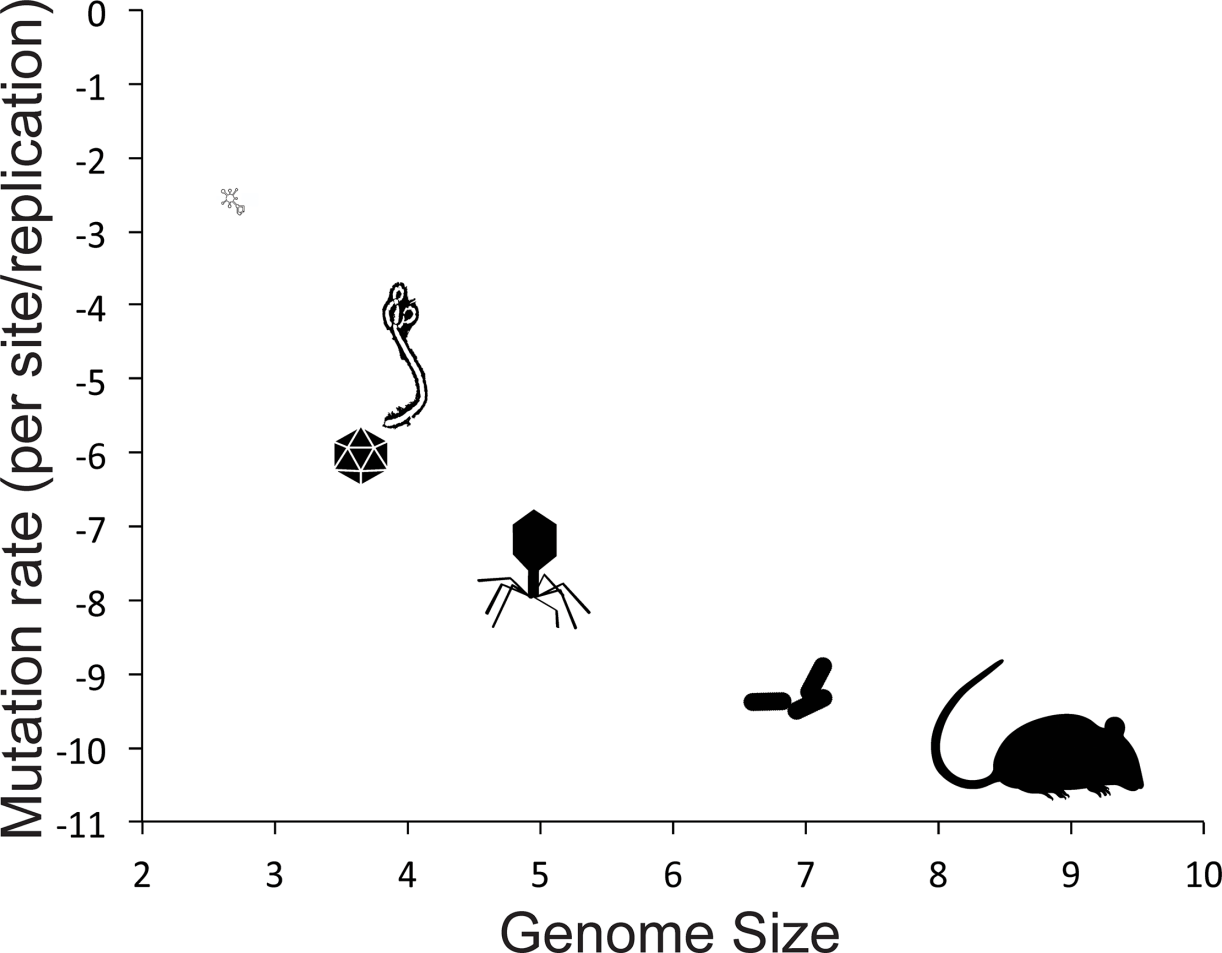
Biological mutation rates summarized from fastest to slowest: Viroid (RNA elements that cause some plant disease without encoding any genes), viruses (RNA shown as Ebola, single-stranded DNA shown as an icosohedron, and double-stranded DNA shown as a myophage), prokaryotes (rod-shaped bacteria), and eukaryotes (rodent). Icons are roughly the size of the range of mutation rates and genome sizes of measured organisms within that group. Axes are log-transformed, data as in [3]. *Images are in the public domain except viroid [4], single-stranded DNA virus (icon made by Pixel perfect,* *www*.*flaticon*.*com**)*, *and rodent (icon made by Freepik*, www.flaticon.com).

## Mutation rates are evolvable and can respond to selection

**Fig 2:**
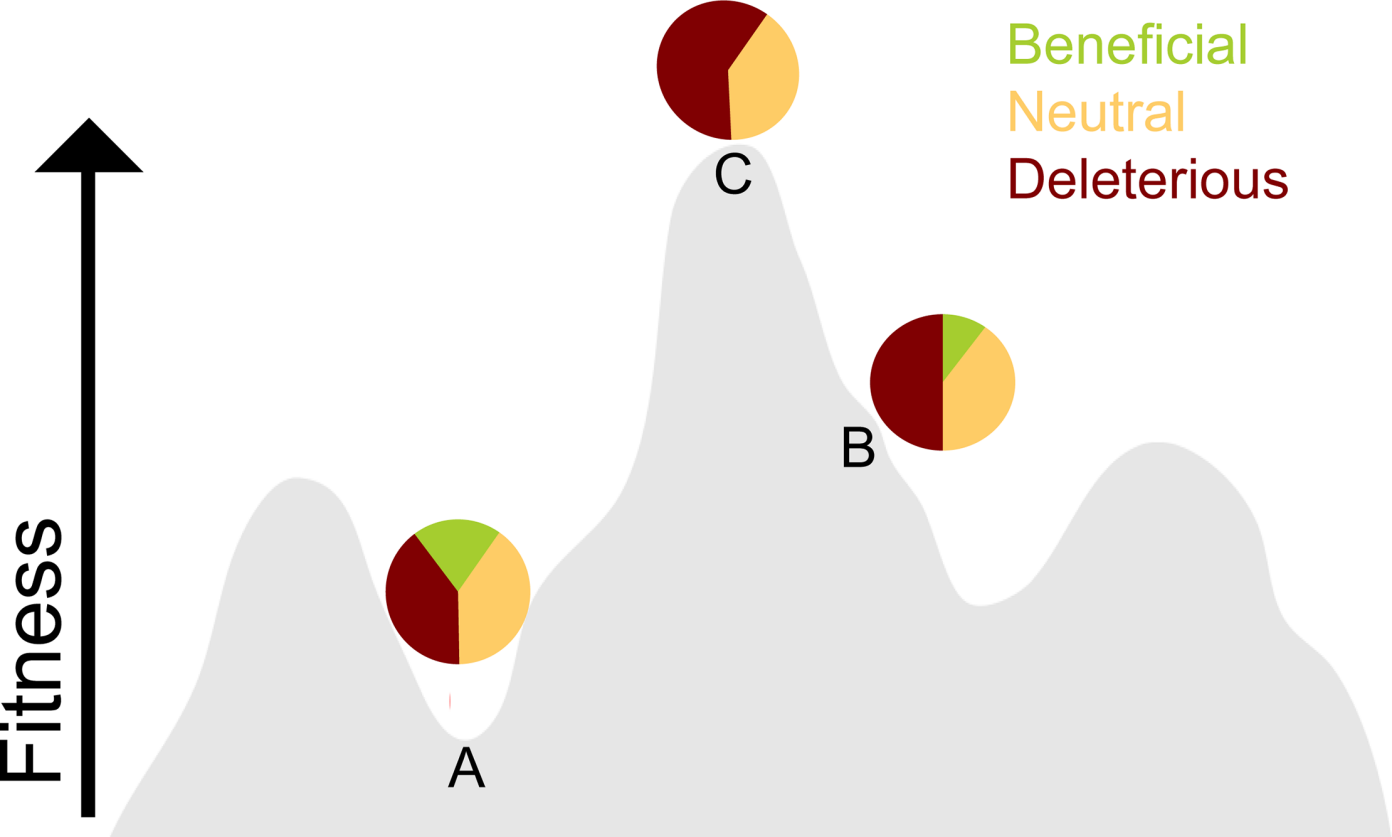
A fitness landscape showing three genotypes on different places on the landscape (A, B, and C) and a schematic pie chart of the distribution of mutations available to each genotype. The genotype at A is not well adapted to the environment (far from a fitness peak) so has a larger fraction of mutations that would be beneficial. The genotype at B is more fit than A and is closer to a fitness peak, so it has a smaller fraction of beneficial mutations than that at A. The genotype at the fitness peak C does not have any way to become more fit on this landscape and thus has no beneficial mutations available to it. The allocations of mutations as beneficial, neutral, and deleterious is for representational purposes only (not based on actual data), and the proportion of neutral mutations was held constant for all three genotypes. *Figure includes a fitness landscape from the public domain*, *originally created by C*. *Wilke*.

In some cases, there is no benefit to mutation at all. At an extreme, an organism that’s “perfectly” adapted to its constant environment would do best to reduce its mutation rate to zero—there are no more beneficial mutations, so all mutations are likely worse than the current genotype (see C in Fig 2). In a constant environment (one where the fitness landscape does not change), it would be best for the optimal genotype to not mutate at all. At another extreme, if an organism is suddenly thrust into an environment that it’s not well adapted to (akin to being at A in Fig 2), there is a larger fraction of potentially beneficial mutations available and having a nonzero mutation rate would be preferable to all descendants always staying exactly the same. The more variable the environments an organism experiences and the lower fitness the organism is in those environments, the more an increased mutation rate would be favored since there is a greater chance per mutation of a mutation being beneficial.

Organisms may not be able to change the fraction of mutations that are deleterious, but they do have some control over their mutation rates, which can limit the number of deleterious mutations that will plague their descendants. Of course, a lower mutation rate comes with the tradeoff that it will also limit the smaller fraction of beneficial mutations—alleles that are beneficial in the current environment and that will help an organism leave more descendants over time. It would also limit the accumulation of neutral (or nearly neutral) variation in populations that might be beneficial if circumstances change, alleles that could be beneficial in a new environment or after climactic change [5]. The mutation rate of all cellular life is under selection, and cells have evolved many ways of tweaking their mutation rates—largely to lower the mutation rate inherent in a fast-moving, processive polymerase replicating their large genomes. These involve proofreading components of the polymerases themselves and a variety of other proteins and systems to check for errors in DNA and to repair common kinds of DNA damage [6]. Some DNA viruses with larger genomes also have DNA repair proteins, and the very largest RNA viruses have some ability to proofread and correct replication errors [7]. Mutant viruses and cells with lowered mutation rates can be isolated by exposing cells or viruses to mutagens, but just as there are proteins and alleles that decrease mutation rates, there are mutations to break those proteins and other alleles that increase mutation rates, which are beneficial in some environments [8].

RNA viruses are perhaps the most intriguing biological entities in which to study mutation rates. They encode their replication machinery, and thus their mutation rates can be optimized for their fitness (in comparison to small DNA viruses that use the polymerases of their host cells). Their inherently high mutation rates yield offspring that differ by 1–2 mutations each from their parent [9], producing a mutant cloud of descendants that complicates our conception of a genotype’s fitness. Their ability to rapidly change their genome underlies their ability to emerge in novel hosts, escape vaccine-induced immunity, and evolve to circumvent disease resistance engineered or bred into our crops [10, 11]. On the other hand, their mutation rates are an exploitable Achilles’ heel: researchers and clinicians can increase RNA virus mutation rates using nucleoside analogues, and a 3–5-fold increase in mutation rate causes lethal mutagenesis in human-infecting viruses like poliovirus and influenza [12, 13]. The exogenous mutagen causes enough additional mutations, which are often deleterious, so that the progeny RNA viruses are of lower fitness, eventually leading to ecological collapse of the population [14]. Another way in which researchers have seen the constraints imposed by the high mutation rate of RNA viruses is in their limited genome size—the mutation rates per nucleotide are too high to increase their genome size without having a higher per-genome accumulation of mutations [9, 15]. Researchers have suggested that RNA virus mutation rates have evolved to be just under the threshold for lethal mutagenesis (sometimes referred to as error threshold [16]) but that selection for genetic diversity and other consequences of a high mutation rate push RNA viruses to near their catastrophic limits. It has been hard to assess this assumption and verify that RNA viruses have their optimal mutation rates due to natural selection on mutation rate.

## Poliovirus mutation rate and fidelity

One of the best-studied systems for RNA virus mutation is poliovirus, in which a now frequently used lower mutation rate mutant (G64S in the 3D RNA-dependent RNA polymerase, 3D:G64S) was characterized, simultaneously, by virologists working at two locations in the San Francisco Bay Area [17, 18]. The 3D:G64S strains not only have a lower mutation rate than wild-type polio but also are less fit in several ways: in one-step growth curves, in cell culture passaging, and in mice, in which they have reduced virulence (the 3D:G64S strains more slowly invade the central nervous system). They are more fit than wild-type poliovirus only in the presence of mutagens, in which their lower mutation rate reduces the inherent number of mutations in each progeny genome, so more exogenous mutations can be tolerated. The 3D:G64S strain also has measurably less genetic diversity during infections, which has suggested a link between population diversity and virulence as well as the adaptability that is conferred by having more standing genetic variation and being able to more rapidly create more variation. However, these conclusions are largely correlational and theoretical, as it has been difficult to conduct experiments to definitively prove that it is indeed the reduced mutation rate of 3D:G64S and not other effects of this mutation causing the reduced virulence and fitness observed in experiments.

In this issue of *PLOS Biology*, Fitzsimmons and colleagues show that reduced replication speed explains more of the effects of the 3D:G64S than its reduced mutation rate per se [19]. There is an intuitive link between replication speed and mutational fidelity [15, 20]—it’s easier for anyone or anything to complete a repetitive task if one can tolerate a certain level of mistakes. If a task is critical to do without any errors at all, it will likely need to be done more slowly with more care and attention. That slower/more accurate relationship has been suggested by previous, less sequencing-intensive work [21], but not all mutations in poliovirus obligately affect both replication speed and mutational fidelity. Fitzsimmons and colleagues demonstrate that a compensatory mutation in 3D:G64S can restore replication speed but not affect the lower mutation rate of 3D:G64S, and this increases viral fitness (2C:V127L). This key experiment teased apart two highly correlated traits to reveal that replication rate affects fitness more than mutation rate.

Further, Fitzsimmons and colleagues cast doubt on the wild type’s advantage of genetic diversity for virulence. The process of entering the mouse central nervous system is a severe bottleneck and is dominated by drift compared to selection—both the wild type and 3D:G64S polioviruses have similar diversities in the mouse central nervous system [19]. Deep sequencing of cell culture-passaged wild type and 3D:G64S populations revealed that both lacked genetic diversity at a meaningful level (SNPS at 0.1%). Finally, the wild type and 3D:G64S increased fitness by identical amounts after passaging in cell culture, refuting that the lower mutation rate of the 3D:G64S strain reduces adaptability. Altogether, this new work suggests that the 3D:G64S strain has a lower fitness because of slower replication, not its reduced mutation rate. RNA viruses like poliovirus likely have higher mutation rates than what would be optimal for the organism because higher mutation rates are, in part, a byproduct of selection for faster genomic replication.

This deeper dive into RNA virus replication fidelity will focus researchers on the consequences of RNA viruses coping with higher than desired mutation rates. This makes the clinical uses of lethal mutagenesis easier to understand—the small increases in mutation rate are not knocking RNA viruses off an optimal peak but are a further insult to an already nearly intolerable mutation rate. Also, just as bacterial populations are known to house mutation rate polymorphisms [22], this work should strengthen the nascent field of understanding mutation rate variation within RNA viral populations [23]. Additionally, replication time (generation time) may be a larger component of understanding virus evolvability than it has been given credit for—likely undervalued because of the difficulties in measuring that trait in multicellular organisms [24, 25].

RNA viruses have high mutation rates, but they may tolerate them rather than revel in them. That they were optimized for genetic variation alone is a “just so story” that should be skeptically re-examined as the more complicated biological reality is revealed [26].
